# Pathogens as Instruments of Murder: The Forensic and Cultural Legacy of Karl Hopf

**DOI:** 10.1097/PAF.0000000000001106

**Published:** 2025-12-22

**Authors:** Ernesto Damiani, Alessia Comacchio

**Affiliations:** * Department of Biomedical Sciences, University of Padova; † INPS, Forensic Medicine Unit for Padua, Padova, Italy

**Keywords:** historical forensic science, Karl Hopf case, biocrime, widal seroreaction, forensic medicine

## Abstract

The Karl Hopf case (1913-1914) represents the first thoroughly documented European instance of biocrimeintentional poisoning, using live bacterial cultures for criminal purposes. This study examines the forensic significance of the case, including the application of microbiological agents as instruments for homicide and the pioneering forensic use of the Widal seroreaction test to establish a causal link between pathogens and victims. The successful detection of arsenic in the cremated remains further expands the scope of postmortem toxicological analysis. The case catalyzed legislative reform in Germany, prompting stricter regulations on the sale and distribution of pathogenic microbes. Beyond its scientific impact, the Hopf case entered psychoanalytic discourse through Freud and Reik’s interpretation of Hopf’s linguistic slip and influenced popular culture, notably Arthur Conan Doyle’s “The Adventure of the Dying Detective.” Unlike other suspected microbial poisoning cases in Europe and the United States, the Hopf case remains the only one to receive sustained and detailed coverage in the criminological and forensic literature. Bridging forensic innovation, legal reform, and cultural reflection exemplifies how a single criminal investigation can redefine evidentiary standards and shape interdisciplinary understanding of crime in the modern era.

## HISTORICAL CONTEXT

In early 20th-century Germany, the Karl Hopf case distinguished itself due to its unprecedented complexity in poisoning methodology, employing a lethal combination of arsenic and pathogenic bacteria. This innovative approach to murder not only challenged existing forensic techniques but also required the development of new, interdisciplinary methods in toxicology and microbiology to unravel the crime. The case marked a shift toward empirically grounded forensic science, exemplified by bacterial fingerprinting through serological testing and arsenic detection in cremated remains. The Hopf case also sparked important discussions about the ethics and legality of certain forensic practices while simultaneously leaving a lasting imprint on both academic medicine and popular culture.

Importantly, the Hopf case introduced the concept of biocrime into the forensic discourse. Biocrime refers to the intentional use of biological agents—such as bacteria, viruses, or microbial toxins—to harm or kill individuals for criminal purposes. Unlike bioterrorism, which is politically or ideologically motivated, biocrime is typically driven by personal motives such as revenge, financial gain, or interpersonal conflict. The Hopf case is the earliest documented example of biocrime, predating modern biosecurity frameworks and underscoring the forensic challenges posed by microbial evidence.

The purpose of this review is to reexamine the Hopf case, shedding light on the aspects that make it a fundamental case for criminalistics and forensic medicine. By analyzing the innovative scientific methods used, the legal and ethical questions raised, and their cultural impact, we aim to show how the Hopf case became a pivotal moment in the evolution of forensic science, bridging psychological interpretations with empirical, laboratory-based investigation.

## THE HOPF CASE

In January 1914, the most sensational criminal trial in early 20th-century Germany unfolded in Frankfurt.^1^ The defendant Karl Hopf, a former pharmacist, fencing instructor, and dog breeder, was accused of poisoning multiple families.^2–4^ Only the most essential points relevant to this topic are summarized here.

Born in 1863 into a merchant family in Frankfurt, Hopf’s early life featured various professional pursuits.^4^ After military service in 1884, he worked in drugstores in London in 1888 and went to Casablanca in 1889, where he returned to Frankfurt in 1891 to recover from malaria, which he had contracted. Later, he returned to Brussels and London, where he performed in vaudeville for the first time.^5^ Returning to Germany in 1894, he gained prominence as a breeder of Saint Bernards.^6,7^

Beneath Karl Hopf’s outwardly respectable persona lay a persistent pattern of concealed criminal behavior. Suspicions regarding his use of the poison surfaced long before his conviction. The death of his father in 1895, characterized by unexplained gastrointestinal symptoms, initially raised concerns, although they were misdirected.^2^ Subsequent fatalities, including those of his child born out of wedlock in 1896 and his first wife, Joséfa Henel, in 1902, provoked unease, yet no legal action ensued. By 1907, formal suspicions emerged concerning the deaths of his wife and child, but the proceedings were discontinued because of insufficient evidence.^4^ Repeated reports and investigations on the deaths of his second wife and daughter were dismissed. It was only following the grave illness of his third wife, Wally Hopf, that authorities intervened; Hopf was arrested on April 14, 1913.^3^ The ensuing investigation uncovered a disturbing and sustained pattern of suspicious deaths and illnesses within his immediate circle, spanning nearly 2 decades.

**FIGURE 1:**
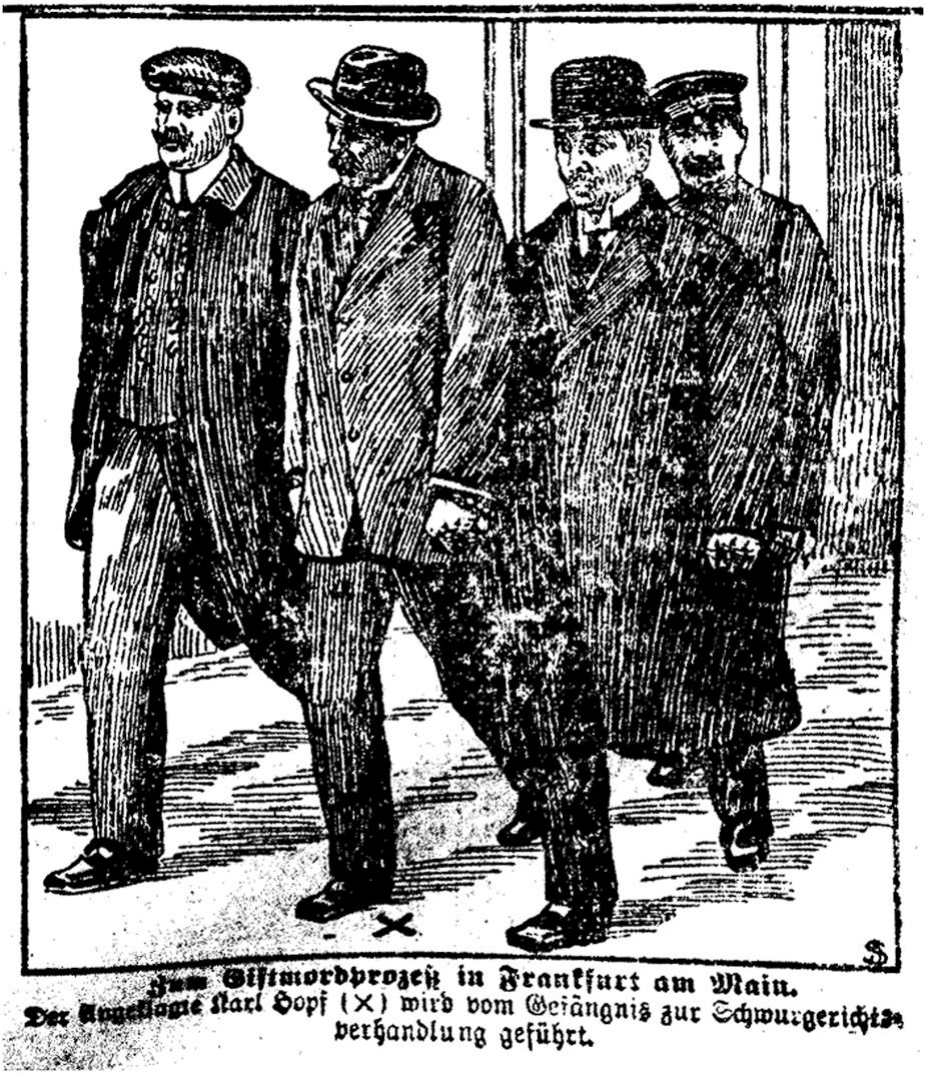
Karl Hopf, the accused in the Frankfurt poison murder trial, is escorted from prison to the jury court hearing. Image source: Anonymous. Der Giftmörder Hopf. *Prager Tagblatt*, January 16, 1914, p. 3. Original German caption: “Zum Giftmordprozeß in Frankfurt am Main. Der Angeklagte Karl Hopf (X) wird vom Gefängnis zur Schwurgerichtsverhandlung geführt.” [The poison murder trial in Frankfurt am Main. The accused Karl Hopf (X) is being led from prison to the jury court hearing].

On January 12, 1914, the trial began shortly after 9 a.m. (Fig. 1). Hopf was charged with a completed poisoning murder in 4 cases (his first wife, Josefa Henel; his daughter, Elsa Hopf; his child born out of wedlock son, Karl Richter; his father, Wilhelm Hopf) and the crime of attempted poisoning murder in 3 cases (his second wife, Christine Schneider; his third wife, Wally Siew; and his mother, Auguste Hopf).^3^

In his 1921 monograph *Psychologie des Giftmordes*, Erich Wulffen^5^ wrote: “The incriminating material that could be presented to Hopf at the very first stage of the proceedings was devastating”. The “devastating material” to which Wulffen was referring included the discovery of arsenic in Hopf’s residence and in the ashes of his mother, the presence of typhoid cultures in his home, and serological findings in his third wife indicating specific antibodies against the same strain—elements that strongly supported the suspicion of deliberate poisoning. “Hopf recognized this with the sharpness of a criminal mind and made a confession. He openly admitted that he had married his third wife solely to kill her—just like the previous ones—and to claim the insurance payout”.^5^ Court records confirm that Hopf admitted to administering cholera, typhus, and arsenic to his wife, and had previously acquired a wide range of lethal substances including typhoid and cholera cultures. These findings, presented in court and supported by forensic analysis, underscore the deliberate and methodical nature of the poisonings.^8^ Although he later attempted to modify his confession, claiming that the idea came after marriage, the damage was complete. This admission became the cornerstone of prosecution, leading to the discovery of other crimes.

German bacteriologist Prof Max Neisser (1869-1938), who served as the court-appointed expert in 1914, reported that the *Salmonella typhi* culture used in the attempted homicide of Hopf’s third wife was received on July 19, 1912.^9^ Notably, Hopf’s personal calendar entry for July 31 included the initials “W” (Wally, his wife’s first name) and “B. Ta.” (*Bacillus typhi abdominalis*, the bacterium responsible for abdominal typhus, that is, typhoid fever), suggesting a premeditation. According to Lempp,^10^ Hopf began infecting his wife—already physiologically compromised by prior administration of arsenic and digitalis—on July 31, 1912, by mixing Salmonella typhi into ground meat.

Hopf’s behavior throughout this period resembled a clinical trial more than a caregiving effort. He meticulously recorded his wife’s temperature beginning August 5, and from August 7 onward, her fever increased with the predictability of a controlled experiment and remained elevated for several weeks.^9^ He subjected his wife to experimental procedures, treating her as a human test subject—a fact documented in contemporary forensic and medical reports. Hopf did not merely attempt murder, but he deliberately monitored the progression of the disease with scientific precision. In so doing, he actively evaluated the efficacy of the poisons he had administered. According to his confession, he also administered cholera bacilli, although his wife did not fall ill, as the culture was no longer virulent.^10^

The jury found the accused guilty of the murder of the first wife and attempted murder of the second and third wives, as well as both children.^5^ He was acquitted of poisoning the father and mother because of a lack of sufficient evidence.^5^ The court sentenced Hopf to death for murder and 15 years in prison for attempted murder.^11^ The verdict recognized both the gravity of the charges and the effectiveness of forensic investigation in proving them, underscoring how modern science could expose even the most medically concealed crimes. Hopf was executed on March 23, 1914.^5^

## KARL HOPF’S USE OF PATHOGENIC BACTERIA AS INSTRUMENTS OF MURDER

The most striking and deeply unsettling aspect of Karl Hopf’s crimes is his calculated use of infectious bacterial cultures as instruments for murder. In a 1913 review article focused specifically on the criminal application of pathogenic microbes, A. Abels^12^ (first name not specified) described Hopf’s case as “the first known and verified case in Europe” where pathogenic bacteria were administered as a means of murder. This biological dimension of the crime not only shocked investigators but also revealed troubling gaps in scientific oversight and biosecurity.

Following renewed allegations, criminal investigators conducted a search of Hopf’s residence and discovered a disturbing array of toxic substances such as arsenic, strychnine, bitter almond water, deadly nightshade extract, foxglove plants, sublimate, morphine, opium, and cyanide.^4,5,9^ The most striking, however, was the presence of 3 viable bacterial cultures, specifically *Vibrio cholerae*, *Salmonella typhi*, and *Clostridium tetani*, while a *Burkholderia mallei* culture no longer contained viable organisms.^9^ The tetanus culture was extremely virulent; the typhoid fever culture also highly virulent, whereas the cholera culture showed no significant toxic effects on animals.^9^ This fully aligns with Hopf’s expressed dissatisfaction regarding their “very poor effect on humans,” which led him to demand replacements.^13^ Indeed, during the trial, Hopf once again cultivated cultures of *Cholera asiatica* in November 1912, which were probably delivered fresh from the theater of the Balkan Wars of 1912-1913 in Bulgaria.^14^

Hopf obtained these cultures from the *Král’sche Sammlung von Mikroorganismen*,^15,16^ a private institution founded in 1890 in Prague by František Král (1846-1911). The collection was aimed at providing a service-oriented repository of microorganisms.^17^ Král maintained correspondence with microbiologists across Europe and the United States, acquiring cultures directly from original researchers. For instance, *Mycobacterium tuberculosis* strains in the Král collection were received directly from Robert Koch.^17^ After Král’s death in 1911, the collection was acquired by Professor Ernst Pribram (1879-1940) and transferred in 1914 to “Staatlichen Institut für die Herstellung von Diphtherieserum” in Vienna. Hopf received a total of 15 shipments from the collection.^15^

To evade institutional scrutiny, Hopf used a falsified letterhead bearing the name of a fictitious scientific institute—the “Chemical, Bacteriological and Therapeutic Institute Hopf.”^2^ This deception was necessary because the distribution of pathogenic microorganisms was strictly limited to universities and specialized bacteriological laboratories.^17^ Before this, Hopf attempted to obtain cultures of cholera, typhoid, and streptococcus from Berlin bacteriologist Max Piorkowski (1859-1937), who refused his request despite offering payment and persuasion.^11^

A search for Hopf’s residence also revealed a substantial library containing an extensive collection of medical and bacteriological studies.^18^ Neisser^9^ remarked that “Hopf’s book knowledge of these matters was considerable.” The library included up-to-date scientific publications, pharmaceutical catalogs, and 2 editions of the Criminal Code, suggesting a keen awareness of their legal implications.

This collection painted a portrait of Hopf as a well-read individual, with a focus on medical science, toxicology, and criminal law, enriching the complex psychological profile presented during the trial.

## FORENSIC BREAKTHROUGH: MICROBIAL POISONING AND THE HOPF CASE

The Hopf case marked a pivotal moment in forensic history, introducing microbial agents as instruments of homicide and challenging conventional definitions of poisoning. As noted by Dracy,^2^ it sparked a legal debate over the boundaries of toxicology: “Should the inoculation of a microbial virus—the germ of typhoid fever or tuberculosis—fall under the category of poisoning?”^2^ Although Karl Lempp (1881-1960) emphasized the difficulty of proving intentional bacterial poisoning,^10^ the forensic literature did not remain silent. Two notable references can be interpreted as affirmative responses to Dracy’s query:

The first appears in Hans Gross’s *Handbuch für Untersuchungsrichter, Polizeibeamte, Gendarmen u. f. m*., ^19^ which recounts a fictional episode set during the 1871 Franco-Prussian War. In this account, a jealous woman disguised as a nurse allegedly transferred purulent material from the septic wound of a deceased patient to the injury of a recovering soldier, intending to cause his death. Although fictional, Gross cited the scenario to illustrate forensic plausibility, demonstrating how microbial transmission could be weaponized in a criminal context.

The second reference comes from Eduard von Hofmann (1837-1897), who coined the term “organized poisons” (*organisirten Gifte*) to describe poisonings caused by pathogens responsible for zymotic diseases, internal parasites (*entozoa*), and other parasitic organisms.^20^ Although Hofmann conceded that the deliberate transmission of such agents was improbable, he asserted that negligent infliction of harm through this means could warrant legal prosecution. This finding reinforces the idea that microbial inoculation, whether intentional or negligent, falls within the legal definition of poisoning.

In 1913, in his review article examining the criminal relevance of disease-causing bacteria,^12^ A. Abels, whose full identity remains unclear, answered Dracy’s question directly by referring to the Hopf case as “the most striking way to show the criminal significance of pathogenic bacteria.”

Collectively, these sources show that forensic literature has already classified bacterial infection as a form of poisoning, with legal consequences for both intentional and negligent transmission.

A second point of forensic interest concerns the possibility of establishing a causal relationship between typhoid fever in the victim and the bacterial strain found in the possession of the accused. In Dracy’s words, “Is there a scientific method to link typhoid fever observed in a patient to the ingestion of a germ from a specific culture?”^2^ This method was first introduced in 1914. Neisser employed the Widal seroreaction test,^2^ a diagnostic technique introduced by Georges-Fernand Widal (1862-1929) in 1896. He demonstrated that the serum of Hopf’s wife agglutinated the *Salmonella typhi* strain found in Hopf’s possession at a dilution of 1:3200, a markedly elevated titer indicative of prior immunologic exposure to that specific bacterial strain. In contrast, the same serum agglutinated typhoid bacilli from other cultures at significantly lower dilutions.^2^ This disparity in reactivity supports the conclusion that the strain recovered from Hopf’s residence was the likely source of infection in the victim, thereby establishing a direct microbiological link between the accused and the disease event.

Notably, this marked the first documented use of the Widal test in a forensic context. Its application in the Hopf case represents a pioneering moment in forensic microbiology, demonstrating that serological methods could be used not only for diagnosis but also as evidentiary tools in criminal investigations involving infectious agents.

Modern techniques, such as polymerase chain reaction (PCR) and whole-genome sequencing, provide high-resolution microbial fingerprinting capable of definitively linking pathogen strains to their sources. One notable example is the 2001 anthrax attack in the United States, in which letters containing *Bacillus anthracis* spores were mailed to various targets. Whole-genome sequencing was used to compare the genetic profiles of anthrax spores with those of known laboratory strains. This allowed them to trace the spores to a specific laboratory source, thereby narrowing the suspected pool.^21^

## FORENSIC BREAKTHROUGH: ARSENIC DETECTION IN CREMATED REMAINS

Arsenic has long held a notorious place in the annals of criminal poisoning, due to its subtle action, easy availability, and the difficulty of early detection. By the early 20th century, it was a well-known toxic agent in forensic medicine. What made the Hopf case exceptional was not the use of arsenic itself, but the forensic breakthrough in detecting it in cremated remains, a challenge previously thought to be insurmountable.

A central toxicological obstacle arose from the analysis of bone ash from Hopf’s mother, who had been cremated. Forensic protocols for thermally altered remains are rudimentary, and cremation is widely believed to destroy or obscure toxicological evidence. This investigation was led by Dr Georg Popp (1861-1943), a pioneering forensic chemist whose earlier work had already advanced this discipline.

Traces of arsenic were also detected in the exhumed bones of Hopf’s first and second wives, both of whom had died under suspicious circumstances. These findings raise the critical question of contamination: whether arsenic could have been introduced postmortem or during burial. To rule out this possibility and establish methodological reliability, Popp and his colleague, Dr Sieber, conducted controlled experiments using canine subjects.^22,23^ Their research yielded 3 critical conclusions:

- Cremation does not artificially introduce arsenic into bone ash, ruling out contamination as a factor.
- Bone ash retains approximately 15% to 17% of the original arsenic content in fresh bone tissue.
- The measured concentration of Hopf’s mother’s bone ash (0.066 mg per 100 g) strongly indicates a fatal ante-mortem dose.^23^

When confronted with evidence of arsenic in the exhumed bodies of his parents, children, and first wife, Hopf offered a series of implausible explanations. He claimed to have recommended arsenic to his 76-year-old mother as a metabolism booster, asserted that he had applied arsenic to his children’s bodies postmortem to prevent decomposition, and alleged that his first wife had used arsenic as a beauty aid.^5^

Although forensic techniques have evolved dramatically over the past century, from rudimentary chemical assays to advanced instrumentation such as inductively coupled plasma mass spectrometry, the results obtained by Georg Popp during the 1914 trial have remained scientifically valid. Modern studies, such as those by Cattaneo et al,^24^ have confirmed the persistence of as in cremated remains and validated the forensic methodology applied during Hopf investigations.

## THE IMPACT OF THE HOPF CASE ON THE PUBLIC AND LEGISLATION

The Karl Hopf case resonated far beyond forensic laboratories and courtrooms; it shocked the public, unsettled scientists, and sparked cultural debate across disciplines. Hopf’s deceitful acquisition of bacterial cultures from institutions such as Professor Pribram’s Vienna Laboratory exposed serious vulnerabilities in scientific oversight and raised urgent legislative questions: How do we safeguard dangerous biological materials?

In the German Reichstag, National Liberal MP Keinath declared, “The Hopf poisoning trial has taught us that trade in cultures of poisonous bacteria must be banned.”^25^ Socialist MP Dr Quarck went further, calling for national and international regulation of pathogenic substances.^26^ Although criminologist Paul Näcke (1851–1913) sought to calm public fear, assuring that domestic institutions had denied Hopf access to virulent strains,^27^ this was only partially true. The Hopf case exposed serious flaws in Germany and Austria’s public health regulations, which focused on preventing accidental transmission.^27^ Before 1917, oversight was minimal: physicians faced few requirements, hospitals were exempt, and sellers of live cultures were not required to verify buyers’ credentials, leaving the system open to abuse.^27^ Austria’s framework mirrored Germany’s strict control of high-risk pathogens, and basic diagnostic procedures required approval. However, it shared the same flaw: no obligation to confirm the qualifications of recipients.

Prompted by the Hopf case, Germany enacted key reforms. On November 21, 1917, the new Federal Council regulations required proof of buyer authorization.^27^ These changes marked a decisive move toward active biosecurity enforcement.

The shockwave generated by the Hopf case also reached the realm of psychoanalysis. In the fourth lecture on the psychology of errors from his *Vorlesungen zur Einführung in die Psychoanalyze* (*Introductory Lectures on Psychoanalysis*), Sigmund Freud (1856-1939) recounted a notable slip of the pen made by a German killer, identified only by the initial “H.”^28,29^ This work conclusively identifies Freud’s anonymous “H.” as Karl Hopf. In fact, the error Freud cited was found in correspondence recovered during the search of Hopf’s apartment, where investigators seized personal letters and laboratory notes.^12^ In one such document, Hopf wrote to the Kral Institute in a tone of complaint, saying the cultures were not virulent enough for humans and requesting stronger strains.^9^ In doing so, he inadvertently wrote “in my experiments on people” instead of “on mice and guinea pigs.”^31^ The original German phrase, *bei meinen Versuchen an Mäusen*, was mistakenly rendered as *bei meinen Versuchen an Menschen*.^30^ This error surfaced during the trial and was introduced as part of evidence of prosecution.^9,27^

According to his theory, Freud interpreted the slip not as mere carelessness but as an unconscious confession of H.’s true criminal intent—a striking real-world validation of psychoanalytic principles. He further argued that such a revelation prompted earlier investigative scrutiny, potentially preventing continuation of the murderer’s activities.

It is noteworthy that the anecdote described by Freud, featuring the German killer “H.” and his revealing slip of the pen, entered the literature of psychoanalytic criminology due to the work of his student, Theodor Reik (1888-1969).^30^ The slip of the pen attributed to Hopf served as a paradigmatic illustration of Reik’s concept of the “third ear”—a heightened receptivity to the latent significations embedded within verbal expression—manifesting as a salient instance of unconscious revelation.^31^

Another testament to the impact of the Hopf case on public discourse can be found in the realm of detective fiction, specifically in Sir Arthur Conan Doyle’s 1913 Sherlock Holmes story, “The Adventure of the Dying Detective.”^32^

In the tale, Holmes feigns a deadly illness—“Tapanuli fever,” a rare tropical disease—to expose the villain Culverton Smith, an amateur bacteriologist who has weaponized pathogens to commit murder. Smith’s character bears a striking resemblance with Hopf. Both men are nonprofessional scientists who have manipulated bacterial cultures for various purposes. Each employed infectious agent is an instrument of murder that exploits their knowledge of microbiology and disease to achieve lethal outcomes. Their methods were steeped in deception, cloaked in the guise of legitimate scientific inquiry, and both chose to kill not through conventional means, but by deliberately exposing their victims to deadly pathogens.

What makes this connection especially compelling is the timing: British newspapers reported extensively on Hopf’s trial and his bacterial arsenal in April 1913.^33,34,35^ Most notably, *The Lancet* covered the case of May 1913,^36^ just months before Doyle’s story was published in late 1913. As a trained physician, Conan Doyle regularly follows the literature published in specialized medical journals.^37^ He also had a deep interest in forensic science, having studied under Sir Robert Christison (1797-1882), a professor of Forensic Medicine at the University of Edinburgh. Conan Doyle was clearly familiar with Christison’s experiments on *postmortem* bruising,^38^ which he directly replicated in “A Study in Scarlet,” where Holmes strikes corpses to study the effects of blows after death.^39^

Given this background, Doyle would have been well-positioned to absorb and adapt contemporary headlines into fiction. While he never explicitly acknowledged Hopf as a source, the parallels were too precise to dismiss. Culverton Smith can be read as a literary echo of Hopf, a fictional embodiment of public anxiety surrounding the misuse of science. Holmes’s triumph over Smith offered readers the reassuring narrative that rationality and forensic acumen could prevail against modern threats, even those cloaked in scientific legitimacy.

Doyle’s practice of drawing inspiration from real-life cases has been well-documented. For instance, “The Problem of Thor Bridge (1922)” was based on a real incident^40^ previously described in 1893 by Gross.^19^

## PATHOGENS AS CRIMINAL WEAPONS BEFORE HOPF

The deliberate use of pathogenic agents by Hopf marked a radical departure from conventional poisoning methods, introducing a microbiological method of homicide that was virtually unprecedented in forensics. Neisser regarded the Hopf case as the only reliably documented attempt at murder using bacteria in Europe.^9^ Abels likewise asserted that no other proven case existed, writing in a footnote: “The Hopf case is the first known and verified instance in Europe where pathogenic bacteria were administered as a means of murder.”^12^

However, the earlier “Buturlin murder case” in St. Petersburg suggests a possible historical antecedent. On May 11, 1910, Lieutenant Vasily Dmitrievich Buturlin, son of General Dmitri Sergeevich Buturlin, died under suspicious circumstances allegedly poisoned by Dr Dimitru Patschenko and his brother-in-law Count Patrick O’Brien de Lacy.^41^ Patschenko initially confessed to administering a lethal dose of diphtheria toxin, which was reported by a bacteriologist in Kronstadtski,^42^ and accused him of orchestrating the crime for financial gain. The trial began in St. Petersburg on January 30, 1911;^41^ however, Patschenko later recanted his confession, claiming that it had been coerced.^43^ Despite asserting that Buturlin had injected himself, both men were convicted: de Lacy received a life sentence, and Patschenko was sentenced to 15 years of hard labor.^44^

Historical research on the Buturlin case is complicated by inconsistent transliterations of Patschenko’s names across sources. German newspapers used “Patschenko”,^41–44^ while Abels^12^ and Lempp^10^ preferred “Pantschenko.” Later studies included variants such as “Panchenko”^45^ and “Pantchenko”.^46^

Unlike Hopf’s trial, which received sustained coverage in the international forensic literature,^2,5,9,12,23,24,27^ the Buturlin case was largely confined to newspaper reports. Fritz Strassmann (1858-1940) briefly mentioned “a recently tried case, in which a Russian doctor allegedly injected a culture of cholera bacteria.”^47^ Abels^12^ referred to it as the “poisoning murder trial of Pantschenko–Buturlin,” without further elaboration. Lempp provided the most detailed account, and communication with the German Consulate in St. Petersburg.^10^

A crucial distinction between Hopf and Buturlin cases is forensic clarity. The Buturlin Trial was marked by conflicting opinions. Abels^12^ noted symptoms resembling cholera; however, conclusive evidence of bacterial poisoning is lacking. Lempp^10^ argued that diphtheria toxin, not cholera, was the true cause. In contrast, Hopf’s use of bacterial agents has been thoroughly documented and is widely accepted in forensics. Abels affirmed that the Hopf case was “the first known and verified case in Europe where pathogenic bacteria were administered as a means of murder,”^12^ and Neisser expanded this claim, suggesting it may have been “the first definitively proven case in the world in which bacteria were used for the purpose of attempted murder”^9^.

Abels^12^ and Lempp^10^ also reported a case from the United States involving Dr Hyde in Kansas City, Missouri. According to a cable telegram cited by “B.Z. am Mittag” on March 7, 1910, and later confirmed by the “Berliner Morgenpost” on May 18, 1910, Hyde allegedly poisoned his wife’s uncle, Colonel Swope, with strychnine. More strikingly, the trial revealed that Hyde attempted to eliminate additional family members, including Swope’s nephew and 8 others, by contaminating household drinking water with typhoid bacteria. Although the case received notable coverage in the German press and was referenced in forensic literature as an early example of suspected microbial poisoning for financial gain, Neisser^9^ questioned the reliability of these reports and noted the absence of detailed documentation in the scientific literature.

In conclusion, while the Buturlin and Hyde cases suggest early attempts at microbial poisoning, both suffer from evidentiary gaps and conflicting results. Only the Hopf case stands as a fully documented and scientifically validated instance of bacterial homicide in forensic and criminalistic literature, establishing it as the definitive reference point for biocrime in early 20th-century Europe.

## PATHOGENS AS CRIMINAL WEAPONS AFTER HOPF

In the years following the Hopf trial, only 2 comparable cases emerged. The 1922 Girard case involved an attempted murder in France using bacterial cultures,^48^ whereas in 1946, a similar incident was documented in Italy, where a medical student allegedly sought to infect his father with typhoid using laboratory-sourced cultures.^49^ Whether these cases were directly inspired by Hopf or not remains speculative. Since then, Stephen Morse’s historical survey has documented several instances of the intentional use of live pathogens, excluding bioterrorism, military applications, and chemical toxicology.^50^ However, only one confirmed criminal case in Europe involved the deliberate use of live bacteria: in 1996, Diane Thompson, a laboratory technician in the United Kingdom, deliberately contaminated pastries with *Shigella dysenteriae* type 2, causing gastrointestinal illness among several coworkers.^50^

The declining interest in using bacterial agents as instruments for crime likely reflects broader epidemiological and medical shifts. As infectious diseases became less prevalent and antibiotic therapy became more widespread, bacteria ceased to be practical tools for homicide. These developments also help explain the fading attention paid to Hopf murder trials in forensic and scientific literature over the following decades. Only recently has the Hopf case regained limited recognition, primarily as a historical precursor to bioterrorism discourse.^38,46^

## CONCLUSION

The Karl Hopf case is a watershed moment in forensic science and criminology, distinguished by several groundbreaking elements. First, it represents the earliest thoroughly documented European instance of deliberate bacterial poisoning, marking the emergence of biocrime as a recognized category within forensic discourse. This novel documented use of pathogenic agents has expanded the conceptual boundaries of toxicology and challenged existing legal frameworks.

Second, the case pioneered 2 critical forensic techniques: the first successful detection of arsenic in cremated remains, previously considered impossible, and the inaugural forensic application of the Widal test to detect specific bacterial antibodies. These innovations have significantly advanced postmortem toxicological analyses and forensic microbiology.

Third, Hopf’s case is unique in its comprehensive documentation and analysis by contemporary forensic experts, establishing new standards of scientific rigor in criminal investigation.

Lastly, the case catalyzed crucial reforms in the regulation of live bacterial cultures, exposing critical biosecurity gaps, and prompting stricter controls.

Taken together, these defining features not only distinguish the Hopf case but also helped shape the trajectory of forensic science, legal practice, and biosecurity policy. Its influence extends to academic discourse and cultural representations, and continues to resonate in modern debates on the intersection of science, ethics, and law in forensic practice.
